# Neurophysiological correlates of anhedonia in feedback processing

**DOI:** 10.3389/fnhum.2013.00096

**Published:** 2013-03-26

**Authors:** Gabry W. Mies, Ivo Van den Berg, Ingmar H. A. Franken, Marion Smits, Maurits W. Van der Molen, Frederik M. Van der Veen

**Affiliations:** ^1^Department of Psychiatry, Erasmus MC, University Medical Center RotterdamRotterdam, Netherlands; ^2^Department of Developmental Psychology, Behavioural Science Institute, Radboud University NijmegenNijmegen, Netherlands; ^3^Institute of Psychology, Erasmus University RotterdamRotterdam, Netherlands; ^4^Department of Radiology, Erasmus MC, University Medical Center RotterdamRotterdam, Netherlands; ^5^Department of Psychology, University of AmsterdamAmsterdam, Netherlands

**Keywords:** depression, anhedonia, fMRI, anterior cingulate cortex, feedback processing

## Abstract

Disturbances in feedback processing and a dysregulation of the neural circuit in which the cingulate cortex plays a key role have been frequently observed in depression. Since depression is a heterogeneous disease, instead of focusing on the depressive state in general, this study investigated the relations between the two core symptoms of depression, i.e., depressed mood and anhedonia, and the neural correlates of feedback processing using fMRI. The focus was on the different subdivisions of the anterior cingulate cortex (ACC). Undergraduates with varying levels of depressed mood and anhedonia performed a time-estimation task in which they received positive and negative feedback that was either valid or invalid (i.e., related vs. unrelated to actual performance). The rostral cingulate zone (RCZ), corresponding to the dorsal part of the ACC, was less active in response to feedback in more anhedonic individuals, after correcting for the influence of depressed mood, whereas the subgenual ACC was more active in these individuals. Task performance was not affected by anhedonia, however. No statistically significant effects were found for depressed mood above and beyond the effects of anhedonia. This study therefore implies that increasing levels of anhedonia involve changes in the neural circuitry underlying feedback processing.

## Introduction

Major depressive disorder (MDD) is a serious mental illness, characterized by at least one of two core symptoms: depressed mood and anhedonia (i.e., the loss of pleasure). MDD affects both affective and cognitive functioning. One of the deficits in MDD in which cognition and affect both play a role is impaired feedback processing. Behavioral studies have shown that depressed individuals are hypersensitive to negative feedback. When they make an error or receive negative feedback on their performance, their subsequent performance deteriorates (e.g., Beats et al., 1996; Elliott et al., 1997; Steffens et al., 2001).

In addition to these aberrant behavioral responses, depressed patients have been found to show an increased electrophysiological response to negative feedback, reflected by the feedback-related negativity (FRN; Tucker et al., 2003; Santesso et al., 2008; Mies et al., 2011b), an event-related brain potential (ERP) component that occurs after receiving negative feedback (Miltner et al., 1997). The FRN is presumed to be generated in the anterior cingulate cortex (ACC) (Ridderinkhof et al., 2004).

The ACC can be divided in two subdivisions: a dorsal part, also known as the midcingulate cortex (MCC), which can be further subdivided into an anterior (aMCC) and posterior (pMCC) part, and a ventral part (ACC), which can be further subdivided into a pregenual (pgACC) and subgenual (sgACC) part (Vogt, 2005; see also Shackman et al., 2011). The aMCC, or more precisely, the rostral cingulate zone (RCZ), has received a lot of attention in the literature on error and feedback processing, since it has repeatedly been found more active during errors, conflict and negative feedback than during correct responses and positive feedback (Ridderinkhof et al., 2004).

In depressed individuals, the MCC, which is thought to be involved in cognitive control, has been found *hypo*active, while brain regions primarily involved in emotion processing, such as the amygdala and sgACC, have been found *hyper*active (Mayberg, 1997, 2003; Davidson et al., 2002; Pizzagalli, 2011). It is therefore thought that the top-down control of the “cognitive” areas over the “affective” areas is disturbed in depression (e.g., Taylor Tavares et al., 2008). This dysregulation appears to persist in fully recovered patients (Hooley et al., 2009), which may make them vulnerable to a relapse. It is, however, possible that this dysregulation is not a *result* of the depression, but *predisposes* an individual to develop a mood disorder such as MDD.

In the present fMRI study, we aimed to identify a relationship between a dysregulated circuit in which the MCC and ACC play a key role, reflected in aberrant feedback processing, and the two core symptoms of depression, depressed mood, and anhedonia. In most studies these symptoms are not separated, although it is known that depressed mood is associated with increased negative affect, while anhedonia is associated with decreased positive affect (Snaith, 1993; Pizzagalli et al., 2005), and that positive and negative affect are two independent constructs (Watson et al., 1988). Anhedonia has been associated with a blunting of behavioral and neural responses to the valence of stimuli (Steele et al., 2007; Dowd and Barch, 2010), whereas depressed mood has been associated with a negativity bias, i.e., the tendency to interpret ambiguous information in a negative way (e.g., Bouhuys et al., 1995). We, therefore, hypothesized that feedback processing would be differentially influenced by anhedonia and depressed mood.

For this purpose we recruited undergraduates who displayed mild depressive symptoms, and let them perform a time-estimation task with two important dimensions of feedback: valence (positive vs. negative feedback) and validity (valid vs. invalid feedback, i.e., feedback that is informative and therefore relevant for behavioral adjustments vs. uninformative/irrelevant feedback). In contrast to most tasks, in which the valence and information value of feedback are highly correlated, this paradigm enables us to disentangle emotion processing (valence processing) from cognitive control (validity processing). We have reported on this task in previous ERP papers including one that involves clinically depressed individuals (Mies et al., 2011b,c). In a previous fMRI study, this time-estimation paradigm showed that the RCZ was primarily sensitive to the validity of the feedback, whereas the pgACC was mainly sensitive to the valence of the feedback (Mies et al., 2011a).

In the present study, we investigated the effects of the core symptoms of depression on these neural correlates of feedback processing. Since anhedonia has been associated with a blunting of neural responses to the valence of stimuli, in e.g., the ventral striatum/nucleus accumbens (Steele et al., 2007; Dowd and Barch, 2010), we hypothesized that anhedonia would be associated with a blunted neural response to the valence of the feedback in this region as well as in the pgACC, i.e., a smaller difference between responses to positive and negative feedback. Depressed mood, on the other hand, was expected to lead to a blunted neural response to the validity of the feedback in the RCZ, i.e., a smaller difference between responses to valid and invalid feedback.

## Materials and methods

### Participants

Participants were recruited by means of advertisements on college-wide electronic bulletin boards of the Erasmus University and the Erasmus MC—University Medical Center Rotterdam. The study was approved by the ethics committee of the Erasmus MC and all participants gave written informed consent. Participants received EUR 25 for participation.

Respondents were asked to fill out the Dutch translation of the Beck Depression Inventory (BDI; Beck et al., 1961; Bouman et al., 1985) assessing depression severity, and a short questionnaire assessing eligibility for participation in an MRI study. The BDI consists of 21 items, each including four statements (ranging from 0 to 3), assessing several symptoms of depression experienced in the last week. High scores indicate more depressive symptoms. In order to obtain a broad range of scores on our symptoms of interest, i.e., depressed mood and anhedonia, we selected participants on the basis of their overall BDI score at screening. Especially those who had a high score (≥10), indicative of mild depressive symptoms (e.g., Bouman et al., 1985) and those who had a low score (<3) at initial screening were invited to participate and were further screened for eligibility. This resulted in a range of BDI scores between 0 and 26 (*M* = 7, *SD* = 7) at the time of scanning, and, importantly, resulted in a broad and continuous range of scores on the questionnaires assessing depressed mood and anhedonia, specifically.

Exclusion criteria were: self-reported neurological illness, severe somatic illness, psychiatric illness other than depression, current treatment for any psychiatric illness (including depression), substance abuse, use of medication which affects the central nervous system (e.g., antidepressants), pregnancy, and any contra-indication for having an MRI-scan. Health criteria were assessed by means of a self-developed questionnaire and contra-indications for MRI were assessed by means of a standard questionnaire from the department of Radiology.

Eventually, 42 healthy volunteers, 26 female, aged between 18 and 32 (*M* = 23, *SD* = 3.5), participated in this study.

### Questionnaires

To specify the core symptoms of depression, we used the Dutch version of the shortened Profile of Mood States (POMS, McNair et al., 1971; Wald and Mellenbergh, 1990) to assess depressed mood, and the Dutch version of the Snaith–Hamilton Pleasure Scale (SHAPS; Snaith et al., 1995; Franken et al., 2007) to assess trait anhedonia.

The visual analog version of the shortened POMS consists of 32 bipolar adjectives to assess current mood. For each pair of adjectives, scores range from 0 to 100, based on how many millimeters from the left participants made a mark on the line. This version of the POMS measures five dimensions: depression, anger, fatigue, tension, and vigor. The dimension “depression” was used as a measure for depressed mood. It consists of 8 items that represents depressed mood including feelings of sadness, unhappiness, hopelessness, loneliness, and worthlessness (Cronbach's alpha = 0.92). Finally, the SHAPS consists of 14 items to be answered on a 1–4 scale, ranging from absolutely agree (1) to absolutely disagree (4) (Cronbach's alpha = 0.83). Higher sum scores indicate higher levels of anhedonia.

### Time-estimation task

**Figure 1:**
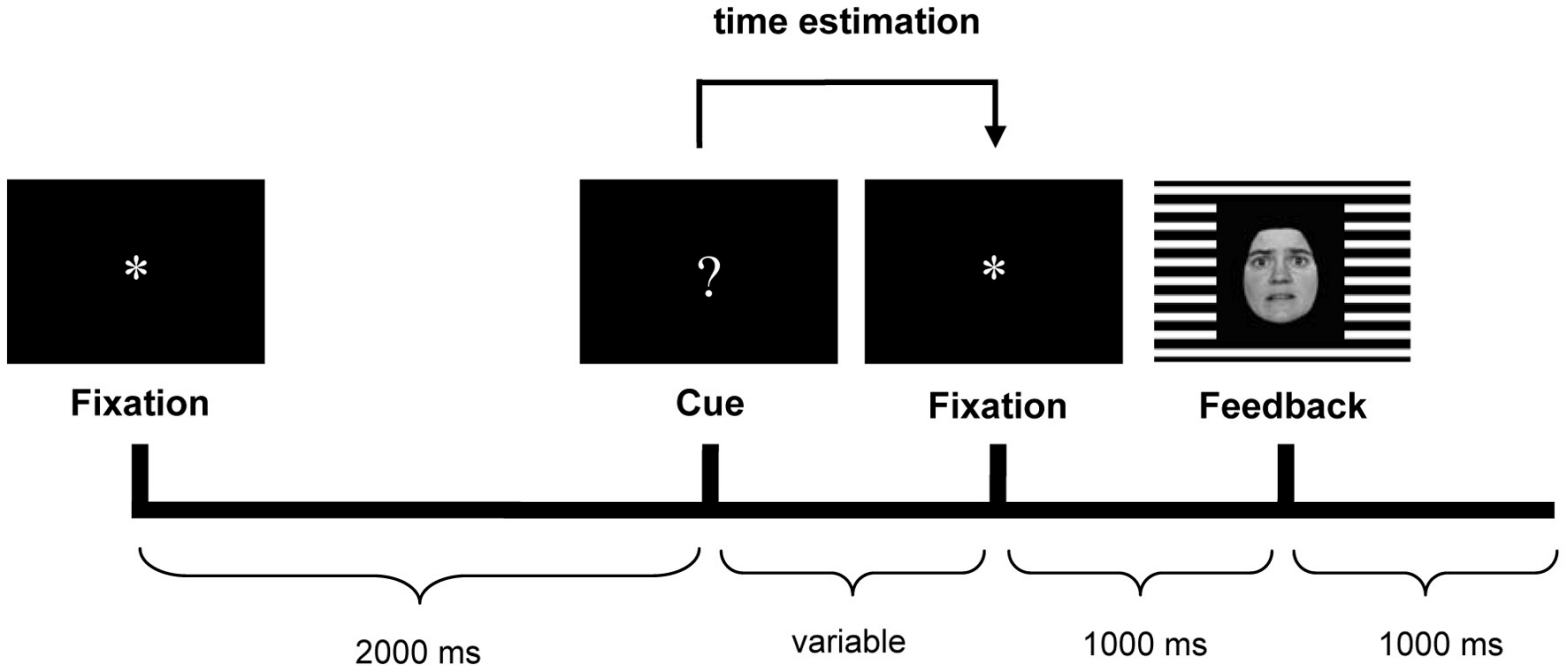
**Trial sequence with an example of the feedback stimulus.** Happy facial expressions indicated positive feedback, fearful expressions indicated negative feedback. The gender (male/female) of the face indicated whether the estimation was too short or too long (counterbalanced across participants). The background grid (horizontal/vertical) indicated whether feedback was valid or invalid (counterbalanced across participants).

The time-estimation task used in the present study was the same as reported earlier (Mies et al., 2011a,c), and was based on the original time-estimation paradigm developed by Miltner et al. (1997). Participants were instructed to produce 1 s intervals. Each trial started with the presentation of an asterisk (“^*^”) in the center of a black screen for 2 s. This asterisk was followed by the cue for estimation: a question mark (“?”), which was replaced with another asterisk (1 s) after the estimation. This second asterisk was followed by the feedback stimulus (1 s) (see Figure 1).

Participants had to indicate the end of the one-second interval by pressing the button of a response device. Following the button press, they received performance feedback, i.e., positive feedback if their response occurred within a specified window around the target (900–1100 ms), and negative feedback if the response occurred outside the window. Unbeknownst to participants, the window was dynamically adjusted on each trial (±20 ms) to ensure an equal amount of positive and negative feedback stimuli (see Miltner et al., 1997).

Estimates were followed by feedback. The feedback consisted of face stimuli presented against a horizontal or vertical background grid. The background grid communicated the validity of the feedback stimulus to the participants (valid vs. invalid). Valid feedback was based on the participant's performance. Invalid feedback was determined randomly by the computer, with a maximum of three invalid feedback trials in a row. Participants received invalid feedback in 50% of the trials. The emotional expression of the face informed participants that their estimate was correct or incorrect (respectively, a happy vs. a fearful face). Finally, in case of incorrect estimates, the gender of the face indicated whether the estimate was too short (e.g., a male face) or too long (e.g., a female face). The faces used in this study were from the Ekman and Friesen pictures set (Ekman and Friesen, 1978).

### Procedure

Participants were seen twice. The first time, participants were asked to fill out the SHAPS and they practiced the two tasks they had to perform in the scanner. The first task participants had to perform was the time-estimation task as described above. Participants were given task instructions and they completed 36 practice trials of the time-estimation task on a computer outside the scanner. The other task was an unrelated task, which is not described in this paper.

Within 4 days of this first meeting (in most instances the next day), participants were scanned. They were asked to abstain from coffee and tobacco for at least 2 h before scanning. Participants first had to fill out the POMS, and were again given task instructions before entering the scanner. When participants were inside the scanner, the visual stimuli were projected on a screen at the end of the scanner bed, which could be viewed by the participant through a small mirror mounted on the head coil. During the time-estimation task participants responded by pressing the button of a response device with their right index finger. Inside the scanner participants performed several practice trials (maximum of 36 trials), after which the first session started, consisting of 120 trials (10 min). After a short break, a second session of the task started which again lasted 10 min. Participants performed 240 trials of the time-estimation task inside the scanner. After these two time-estimation sessions a structural scan was obtained, which lasted about 5 min.

### Magnetic resonance imaging data acquisition

Blood oxygen level-dependent (BOLD) fMRI data were acquired on a 3T GE Healthcare (Milwaukee, WI) scanner. For the functional scans a single-shot gradient echo echo-planar imaging (EPI) sequence was used. The T2^*^-weighted images were acquired in 26 axial slices (thickness = 3.5 mm, interslice gap = 0.5 mm) with a repetition time (TR) of 2000 ms, echo time (TE) of 30 ms, field of view (FOV) of 220 mm, and voxels of 1.72 × 1.72 × 3.50 mm. The interval between trials was about 5 s. In each session of 120 trials 310 volumes (8060 functional images) were obtained. In addition, five dummy scans were made before the task started in order to obtain a steady-state magnetization.

For anatomical reference, a 3D high-resolution inversion recovery fast spoiled gradient recalled echo T1-weighted sequence was used, which covered the whole brain. One hundred and ninety-two slices were acquired with an effective slice thickness of 0.8 mm, FOV of 250 mm, and voxels of 0.49 × 0.49 × 0.80 mm.

For pre-processing and processing of the fMRI data SPM5 (Statistical Parametric Mapping, Wellcome Trust Centre for Neuroimaging, University College London, UK) was used. Preprocessing of the structural data included manual reorienting, unified segmentation using the Montreal Neurological Institute T1 ICBM template for European brains for gray matter, white matter, and CSF, and normalization using the parameters derived from unified segmentation. Preprocessing of the functional data included manual reorienting, slice time correction, realignment using the middle slice as a reference, and unwarping, co-registration (functional images were co-registered to the gray matter structural image derived from unified segmentation), normalization using the parameters derived from unified segmentation, and smoothing using a Gaussian kernel of 8 mm full width at half maximum, and a high-pass filter of 128 s for temporal smoothing.

### Statistical analyses

Performance data were analyzed by partial correlation analyses assessing the relation between POMS-depression scores and the percentage of correct adjustments after valid negative feedback and the percentage of “correct” adjustments after invalid negative feedback, while correcting for SHAPS-anhedonia scores, and vice versa (examining the relation between SHAPS scores and performance, while correcting for POMS scores).

For the fMRI analyses, a model was made in which the preprocessed fMRI data were coupled to the vectors of feedback onset of each condition (valid positive feedback, valid negative feedback, invalid positive feedback, and invalid negative feedback) in both task sessions. Then two t-contrasts were computed that were used for the whole-brain analyses only: positive—negative feedback (main effect of valence), and valid—invalid feedback (main effect of validity). The individual contrast images resulting from these contrasts were used in a second-level whole-brain analysis.

Whole-brain analyses were performed on the two contrasts. The POMS-depression score and the SHAPS-anhedonia score were added as covariates of interest. The POMS and SHAPS scores were both normally distributed, and were centered by the method of Delaney and Maxwell (1981): the mean of all participants was subtracted from individual scores. Significant voxels and clusters are reported as significant if *P* < 0.05 corrected with the family-wise error (FWE) approach. The Automated Anatomical Labeling (AAL) atlas (Tzourio-Mazoyer et al., 2002) was used to label the significant clusters and voxels.

**Figure 2:**
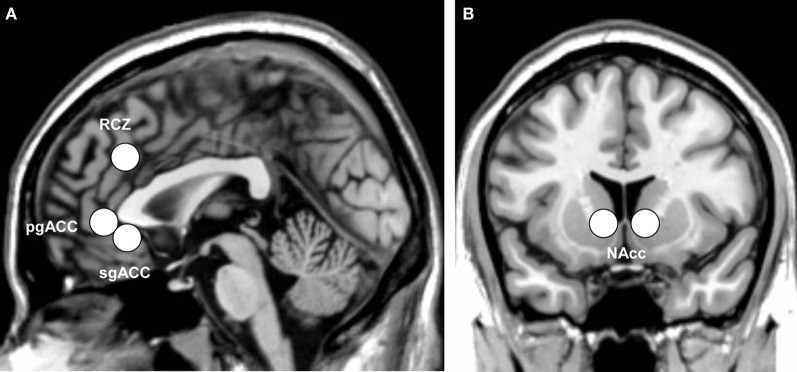
**Indication of the anatomical location of (A) the three subdivisions of the anterior cingulate cortex: the rostral cingulate zone (RCZ), pregenual anterior cingulate (pgACC), and subgenual anterior cingulate (sgACC), and (B) the bilateral nucleus accumbens (NAcc), displayed on the ch2 template of MRIcron**.

Of main interest were, however, the region-of-interest (ROI) analyses. Four ROI analyses were performed using MarsBaR 0.41 (Brett et al., 2002). The left and right RCZ [8 mm sphere around ±8, 30, 32; coordinates adopted from Mars et al., 2005 and implemented in the AAL map of MarsBaR (Tzourio-Mazoyer et al., 2002)], the pgACC [8 mm sphere around 0, 40, −2; coordinates adopted from Nieuwenhuis et al. (2005)], the sgACC [8 mm sphere around 1, 32 −6; coordinates adopted from Matthews et al. (2009)], and the nucleus accumbens [NAcc, ±10, 12, −2, coordinates adopted from Knutson et al. (2008)] were defined as ROIs. Figure 2 illustrates the ROIs examined.

For the ROI analyses, beta-values were extracted from the fMRI data for each feedback condition (valid positive feedback, valid negative feedback, invalid positive feedback, and invalid negative feedback) separately. For each ROI, the extracted beta-values of each participant were exported to SPSS, and subsequently analyzed using valence (positive or negative feedback) and feedback-validity (valid or invalid feedback) as within-subjects factors in repeated-measures ANOVAs with mean-centered POMS-depression and SHAPS-anhedonia scores simultaneously added as covariates. Effects of lateralization in the RCZ and NAcc are not reported.

## Results

One student did not perform according to task instructions, and was excluded from all analyses. Therefore, 41 participants were included in the analyses.

POMS-depression scores ranged from 8 to 457 (*M* = 187, *SD* = 96), and SHAPS scores ranged from 14 to 34 (*M* = 22, *SD* = 5). The POMS-depression scores and SHAPS-anhedonia scores correlated positively with each other (*r* = 0.47, *P* = 0.002).

### Behavioral results

Participants had a mean estimation time of 1053 ± 87 ms, and, as expected, they adjusted their behavior more often in response to valid negative feedback than to invalid negative feedback (85 ± 8% vs. 52 ± 6%). The partial correlation analyses showed a marginal negative correlation between depressed mood and percentage of correct behavioral adjustments after valid negative feedback (*r* = −0.28, *P* = 0.086), indicating that participants with higher scores of depressed mood performed slightly worse after receiving valid negative feedback. No associations were found with anhedonia.

### Whole brain analyses

The results of the whole brain analyses are shown in Tables 1, 2. At the FWE-corrected threshold of *P* < 0.05, all contrasts revealed significant activation patterns, except negative feedback minus positive feedback. Importantly, the whole brain analyses did not reveal any significantly different activation patterns for participants with higher levels of depressed mood or anhedonia.

**Table 1:** **Whole brain analysis for the contrast positive feedback—negative feedback**.

**Area**	**L/R**	**BA**	**Cluster size**	***Z***	**MNI coordinates**		
					***x***	***y***	***z***
**POSITIVE FEEDBACK > NEGATIVE FEEDBACK**							
Insula/Putamen	L		830	6.92	−26	10	−12
Putamen	L		^a^	6.58	−26	−4	4
Putamen	L		^a^	6.56	−22	−6	16
Orbital medial frontal gyrus	R	10/11	584	6.82	4	54	−8
Medial frontal gyrus/Anterior cingulate	L	10	^b^	6.76	−8	48	2
Orbital medial frontal gyrus	L	11	^b^	6.54	−6	56	−10
Putamen	R		671	6.72	24	−8	12
Putamen	R		^c^	6.66	28	12	−10
Putamen	R		^c^	6.59	24	6	0
Putamen	R		^c^	6.40	26	−2	8
Precuneus/Posterior cingulate	L	23/31	153	6.46	−4	−56	24
Precuneus/Posterior cingulate	R	23	^d^	6.10	6	−50	26
Inferior occipital gyrus	R	18	31	6.37	28	−92	−2
Superior frontal gyrus	L	32	79	6.23	−16	36	42
Superior frontal gyrus	L	9	^e^	6.21	−22	26	40
Paracentral lobule	L		17	6.23	−14	−28	54

The Automated Anatomical Labeling (AAL) atlas (Tzourio-Mazoyer et al., 2002) and MRIcron (Rorden et al., 2007) were used to label the significant clusters and voxels. In some cases the nearest gray matter is shown. XjView (http://www.alivelearn.net/xjview) was used to further specify these brain regions when necessary.

a,b,c,d,e Local maximum within the cluster described in the previous line, i.e., a, b, c, d, and e, respectively (p < 0.0001, FWE-corrected). To simplify only the significant activations at the more conservative FWE-corrected threshold of p < 0.0001 are shown.

**Table 2:** **Whole brain analysis for the contrasts valid—invalid feedback, and invalid—valid feedback**.

**Area**	**L/R**	**BA**	**Cluster size**	**Z**	**MNI coordinates**		
					**x**	**y**	**z**
**VALID FEEDBACK > INVALID FEEDBACK**							
Insula	L	47	341	4.62	−30	18	0
Orbital inferior frontal gyrus	R		338	4.60	32	24	−6
Precentral gyrus	L	6	333	4.58	−54	4	18
Inferior parietal gyrus	L	40	740	4.56^*^	−46	−46	42
Middle frontal gyrus	R	46	296	4.56^*^	46	48	6
Inferior parietal gyrus	R	40	562	4.46^*^	52	−40	54
Caudate	R	25	549	4.37^*^	10	18	0
Mid cingulate	R	32	338	4.17^*^	4	26	40
Caudate	L	25	460	3.98^*^	−8	16	−2
**INVALID FEEDBACK > VALID FEEDBACK**							
Middle temporal gyrus	R	39	1212	6.39	52	−62	20
Middle frontal gyrus	L		1511	5.76	−26	26	34
Medial superior frontal gyrus	L	10	^a^	4.93	−6	56	18
Calcarine sulcus	L	17	3947	5.06	−14	−62	16
Superior parietal gyrus	R	5	^b^	4.95	18	−50	60
Precuneus	L		^b^	4.74	−8	−46	46
Superior temporal gyrus	R	42	2686	4.78	54	−30	18
Superior temporal gyrus	R	42	^c^	4.72	56	−28	14
Middle temporal gyrus	L	39	1046	4.78	−48	−68	22
Middle frontal gyrus	R	9	675	4.57^*^	30	30	36
Lingual gyrus	R	30	450	4.38^*^	10	−52	8
Superior temporal gyrus	L	41	1032	4.32^*^	−50	−32	20

The AAL atlas and MRIcron were used to label the significant clusters and voxels. In some cases the nearest gray matter is shown. XjView was used to further specify these brain regions when necessary.

a,b,c Local maximum within the cluster described in the previous line, i.e., a, b, and c, respectively (p < 0.05, FWE-corrected).

* Significant at cluster level only (p < 0.05, corrected).

### Region-of-interest analyses

#### General task effects

**Figure 3:**
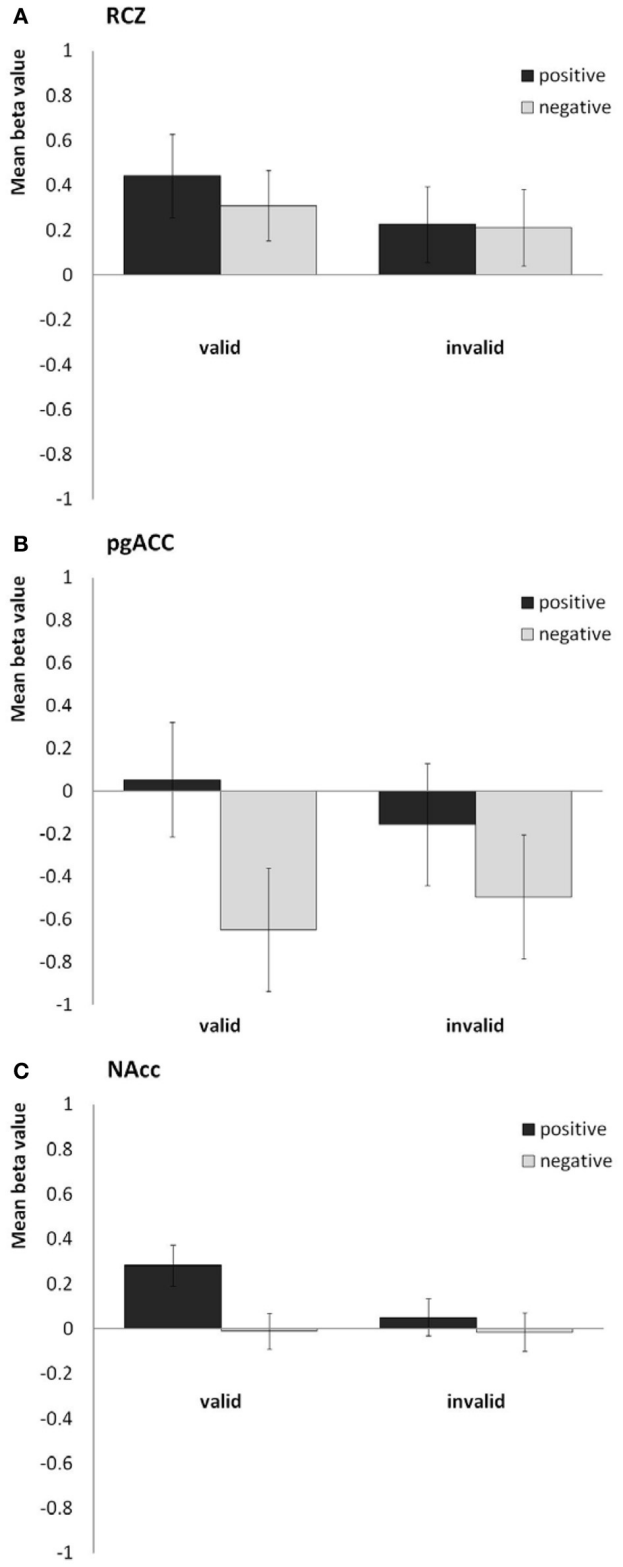
**Bar plots showing the mean beta values for the four predictors (valid positive, valid negative, invalid positive, and invalid negative) for (A) the rostral cingulate zone (averaged across the right and left hemisphere), (B) the pregenual anterior cingulate, and (C) the nucleus accumbens (averaged across both hemispheres)**.

In line with our previous study, we found the RCZ more active in response to *valid* feedback than in response to *invalid* feedback [*F*_(1, 40)_ = 8.1, *P* = 0.007, η^2^_*p*_ = 0.17; see Figure 3A], whereas the pgACC was more active in response to *positive* feedback than in response to *negative* feedback [*F*_(1, 40)_ = 30.4, *P* < 0.001, η^2^_*p*_ = 0.43; see Figure 3B]. The NAcc was more active in response to valid feedback than in response to invalid feedback [*F*_(1, 40)_ = 17.5, *P* < 0.001, η^2^_*p*_ = 0.30], and was more active in response to positive feedback than in response to negative feedback [*F*_(1, 40)_ = 23.6, *P* < 0.001, η^2^_*p*_ = 0.37]. In addition, valence and validity interacted in the NAcc [*F*_(1, 40)_ = 15.8, *P* < 0.001, η^2^_*p*_ = 0.28]; the effect of valence was strongest in the valid condition (see Figure 3C). No interaction between valence and validity was found in the RCZ or pgACC. Finally, the sgACC did not respond differently to the different types of feedback.

#### Effects of depressed mood and anhedonia

The purpose of this study was to examine the separate influences of depressed mood and anhedonia on the four predefined ROIs in relation to these task effects. No statistically significant effects were found for depressed mood above and beyond the effects of anhedonia. Depressed mood only marginally interacted with the effect of validity in the RCZ [*F*_(1, 38)_ = 3.1, *P* = 0.084, η^2^_*p*_ = 0.08], and with the effect of valence in the NAcc [*F*_(1, 38)_ = 3.1, *P* = 0.084, η^2^_*p*_ = 0.08].

**Figure 4:**
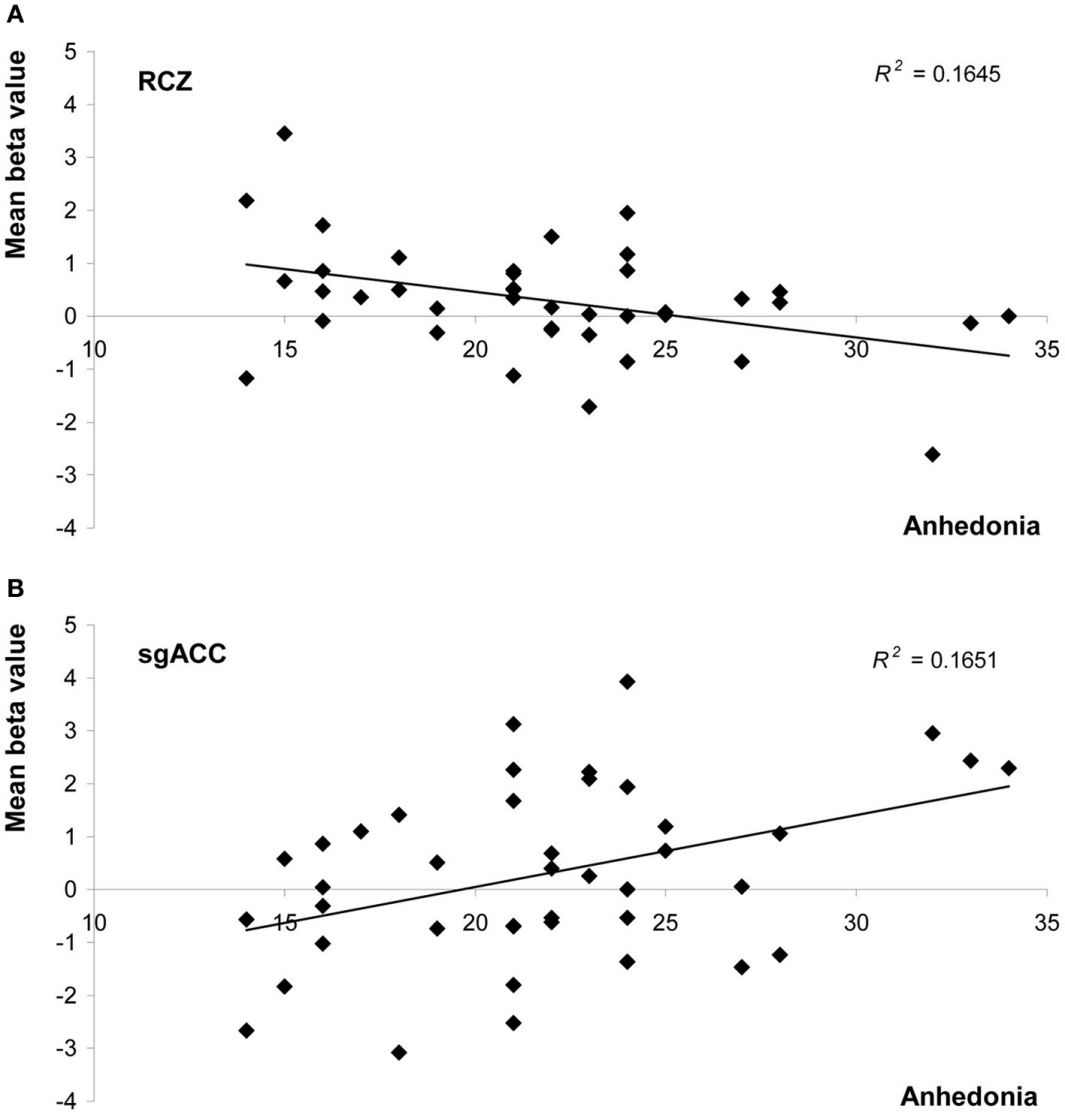
**Scatter plots of mean beta values representing activity in (A) the rostral cingulate zone (averaged across both hemispheres), and (B) the subgenual anterior cingulate as a function of anhedonia (measured with the SHAPS).** Both graphs show values uncorrected for levels of depressed mood.

Anhedonia, on the other hand, did influence feedback processing. In the RCZ we found a main effect of anhedonia, after correction for variation in depressed mood [*F*_(1, 38)_ = 7.5, *P* = 0.010, η^2^_*p*_ = 0.16]. Higher levels of anhedonia were associated with decreased activity in the RCZ, independent of task condition (Figure 4A). Also in the sgACC a main effect of anhedonia was found. In this area higher levels of anhedonia were associated with increased activity, independent of task condition [*F*_(1, 38)_ = 4.2, *P* = 0.048, η^2^_*p*_ = 0.10; Figure 4B]. In the pgACC we found a marginal interaction between anhedonia and valence: more hedonic individuals showed a slightly larger difference between positive and negative feedback [*F*_(1, 38)_ = 3.7, *P* = 0.064, η^2^_*p*_ = 0.09]. Surprisingly, no relationship was found between anhedonia and activity in the NAcc.

## Discussion

The current study investigated the relationship between the two core symptoms of depression, i.e., depressed mood and anhedonia, and behavioral and neural responses to valid and invalid positive and negative feedback. In this student sample, after correcting for the influence of anhedonia, no statistically significant effects were found of depressed mood on feedback processing. Anhedonia, on the other hand, was negatively correlated with activity in the RCZ, and positively correlated with activity in the sgACC in response to feedback stimuli in general, after correcting for the influence of depressed mood (see Table 3). Anhedonia did not affect the behavioral responses to feedback.

**Table 3:** **Overview of ROI findings**.

**ROI**	**Task effects**			**Main effects of depressive symptoms**	
	**Validity**	**Valence**	**Validity × Valence**	**Depressed mood**	**Anhedonia**
RCZ	✓	✗	✗	✗	↓
pgACC	✗	✓	✗	✗	✗
sgACC	✗	✗	✗	✗	↑
NAcc	✓	✓	✓	✗	✗

*RCZ, rostral cingulate zone; pgACC, pregenual anterior cingulate cortex; sgACC, subgenual anterior cingulate cortex; NAcc, nucleus accumbens*; ✓, *statistically significant effect*; ✗, *no statistically significant effect; ↓, decreased activation; ↑, increased activation.*

The general task effects are in line with our previous studies on feedback processing using this time-estimation task (Mies et al., 2011a,c). At the behavioral level, participants performed according to task instructions: they adjusted their behavior more often in response to valid negative feedback than in response to invalid negative feedback. At the neural level, we again found the RCZ to be sensitive to the validity of the feedback and the pgACC to be sensitive to the valence of the feedback, while the NAcc was sensitive to both valence and validity. The sgACC was neither sensitive to the valence, nor to the validity of the feedback, which implies that it does not play a major role in feedback processing.

Anhedonia was associated with an overall decrease of RCZ-activity in response to feedback. This effect was not hypothesized. Recently, Shackman et al. (2011) postulated the “adaptive control hypothesis,” which suggests that the MCC, in particular the RCZ, uses information with a negative value (punishment, pain, and more abstract forms of negative feedback) to bias responding when the most adaptive course of action is uncertain, and therefore integrates emotion, pain and cognitive control. Our findings are not completely in line with this hypothesis, since no effect of valence was found in the RCZ. This finding implies that the RCZ does not only integrate *negative* information with cognitive control, but *positive* information as well. In line with the adaptive control hypothesis, however, is our finding that RCZ activity was influenced by anhedonia, which implies that the RCZ does integrate emotional information, albeit on another level than on stimulus level. Another model of cingulate function, the “predicted response-outcome” (PRO) model, was recently developed by Alexander and Brown (2011), and suggests that activity in the medial PFC (including the RCZ) reflects a learned prediction of the probability and timing of all possible outcomes of an action. According to this model, mPFC/RCZ activity reflects the unexpected non-occurrence or unexpected occurrence of an outcome. This model, therefore, can account for both positive and negative feedback eliciting an RCZ response. Perhaps the general decrease of activity in this brain region in more anhedonic individuals reflects weaker outcome predictions in these individuals, in line with the lack of interest and motivation associated with anhedonia.

Anhedonia was also associated with increased sgACC activity in response to feedback, independent of the type of feedback. A hyperactive sgACC has been rather consistently found in clinically depressed patients (Mayberg, 1997, 2003; Davidson et al., 2002; Pizzagalli, 2011), in healthy persons with high levels of neuroticism or negative affect (Zald et al., 2002; Haas et al., 2007), and in healthy persons subjected to negative mood induction (Mayberg et al., 1999; Berna et al., 2010). This increased sgACC response in combination with a decreased RCZ response implies an imbalance in individuals with higher levels of anhedonia in the neural circuit in which the cingulate cortex plays a key role.

We further expected that higher levels of anhedonia would be associated with a reduced effect of valence in the pgACC and the NAcc. This expected blunted response to positive and negative feedback only marginally reached significance in the pgACC. Surprisingly, in contrast to most studies (Steele et al., 2007; Pizzagalli et al., 2009; Dowd and Barch, 2010), anhedonia was not associated with a blunted response in the NAcc. The discrepancy may lie in the fact that these other studies included clinically depressed or schizophrenic patients in which levels of anhedonia are likely to be higher than in our undergraduates with mild symptoms.

Surprisingly, no significant effects were found of depressed mood on feedback processing, after correction for the influence of anhedonia. We would like to note, however, that there were some trend-level effects, which might be of interest to more closely examine in future studies. Depressed mood appeared to be slightly associated with reduced sensitivity of the RCZ to the validity of feedback. This blunted response to feedback-validity was also expressed at the behavioral level. It should be noted that clinically depressed subjects in a previous ERP study also showed slightly impaired performance after valid negative feedback during the same task (Mies et al., 2011b). These findings suggest that the evaluation of the relevance of the feedback is reduced in individuals with higher levels of depressed mood.

There are several limitations that need to be addressed. First, as expected, anhedonia and depressed mood were correlated, making it difficult to completely disentangle their unique contributions to the present results. When correcting effects of depressed mood for anhedonia, effects did not reach significance, e.g., the interaction between depressed mood and validity of feedback in the RCZ. Although it is unnatural to select participants on the basis of distinct depressed mood and anhedonia scores, it might be useful in future studies to do so. In addition, larger sample sizes may be effective in disentangling their unique effects. Second, we used emotional faces as feedback stimuli, which may cause some concern about whether found effects are due to feedback processing or to emotional face processing *per se*. Emotional faces are differently processed by depressed than by healthy individuals, that is, depressed individuals show enhanced *recognition* and *recollection* of negative emotional expressions such as sadness, and impaired recognition of—or an attentional bias away from—positive expressions (see Leppanen, 2006 for a review). However, we deliberately chose to use easily distinguishable emotional expressions to increase the ecological validity of the feedback stimuli (see also Mies et al., 2011b). By including a valid and invalid feedback condition, we were able to control for the effect of emotion, which is underscored by the RCZ findings. Unfortunately, because the sgACC and pgACC were not sensitive to the validity of feedback, we cannot directly conclude that these areas are responsive to the valence of the feedback rather than to emotion processing *per se*. On the other hand, one could argue that (aberrant) processing of feedback-valence is inherent to (aberrant) emotion processing.

In conclusion, our findings suggest that anhedonia, rather than depressed mood, affects feedback processing at the neural level. Anhedonia was associated with a decreased response of the RCZ to feedback and with an often-reported hyperactive sgACC in depression. Our results imply that increasing levels of anhedonia involve changes in the neural circuitry underlying feedback processing. These atypical neural responses might render subjects vulnerable to depression.

### Conflict of interest statement

The authors declare that the research was conducted in the absence of any commercial or financial relationships that could be construed as a potential conflict of interest.
